# Safety Assessment of Starch Nanoparticles as an Emulsifier in Human Skin Cells, 3D Cultured Artificial Skin, and Human Skin

**DOI:** 10.3390/molecules28020806

**Published:** 2023-01-13

**Authors:** So-Yeon Kim, Hye-Young Shin, Jong-Yea Kim, Se Jin Park

**Affiliations:** 1 School of Natural Resources and Environmental Sciences, Department of Food Biotechnology and Environmental Science, Kangwon National University, Chuncheon 24341, Republic of Korea; 2 Department of Food Science and Biotechnology, Kangwon National University, Chuncheon 24341, Republic of Korea; 3 Agriculture and Life Science Research Institute, Kangwon National University, Chuncheon 24341, Republic of Korea

**Keywords:** emulsifier, starch nanoparticles, 3D cultured skin, cell viability, human skin

## Abstract

Emulsion systems are widely used in various industries, including the cosmetic, pharmaceutical, and food industries, because they require emulsifiers to stabilize the inherently unstable contact between oil and water. Although emulsifiers are included in many products, excessive use of emulsifiers destroys skin barriers and causes contact dermatitis. Accordingly, the consumer demand for cosmetic products made from natural ingredients with biocompatibility and biodegradability has increased. Starch in the form of solid nanosized particles is considered an attractive emulsifier that forms and stabilizes Pickering emulsion. Chemical modification of nanosized starch via acid hydrolysis can effectively provide higher emulsion stability. However, typical acid hydrolysis limits the industrial application of starch due to its high time consumption and low recovery. In previous studies, the effects of starch nanoparticles (SNPs) prepared by treatment with acidic dry heat, which overcomes these limitations, on the formation and stability of Pickering emulsions were reported. In this study, we evaluated the safety of SNPs in skin cell lines, 3D cultured skin, and human skin. We found that the cytotoxicity of SNPs in both HaCaT cells and HDF cells could be controlled by neutralization. We also observed that SNPs did not induce structural abnormalities on 3D cultured skin and did not permeate across micropig skin tissue or human skin membranes. Furthermore, patches loaded with SNPs were found to belong in the “No irritation” category because they did not cause any irritation when placed on human skin. Overall, the study results suggest that SNPs can be used as a safe emulsifier in various industries, including in cosmetics.

## 1. Introduction

In emulsion systems, finely divided solubilized hydrophobic materials are dispersed in an aqueous medium [1,2]. This system typically comprises a mixture of oil and water to facilitate the supply of antioxidants or functional lipid compounds. Because of these properties, emulsion systems are actively applied in the cosmetic, pharmaceutical, medical, and food industries [3]. However, emulsion systems can separate over time, returning to their original state, because the contact between oil and water is inherently unstable. To inhibit separation, a surfactant that stabilizes the emulsion is essential [4]. Although synthetic surfactants are applied in the cosmetic industry, their biosafety is not guaranteed [5]. Several studies have reported that surfactants such as sodium chloride, panthenol, and glycerol included in cleansing products can cause pH changes, barrier dysfunction, and itching in the stratum corneum [6,7]. Accordingly, consumers are demanding cosmetic products made from natural ingredients with biocompatibility and biodegradability. This demand has been partly addressed by the substitution of synthetic ingredients with natural alternatives.

Starch is considered an effective alternative for emulsion stabilization, not only because it is safe due to its nonallergenic nature but also because it is abundant and inexpensive [8]. Natural starch granules may have reduced effectiveness as emulsion stabilizers due to their size and hydrophobicity, but chemically modified nanosized starch can be an effective stabilizer [9]. Nanoparticles can be used as solid stabilizers, providing additional advantages, such as higher emulsion stability [10]. This is called a Pickering emulsion, in which nanoparticles are responsible for forming and stabilizing the emulsion [11]. Thus, various methods, such as acid hydrolysis, are used to reduce the particle size of starch.

Typical acid hydrolysis is time consuming and has low recoveries, which limits the industrial application of starch [12]. In previous studies, the effects of starch manufactured by overcoming these limitations of their physical properties and the formation and stability of Pickering emulsions were reported [13]. In fact, starch nanoparticles (SNPs) manufactured via dry heating under mildly acidic conditions showed a high emulsification capacity and were stable even during heating and freeze–thaw treatment [10,13]. Based on this, SNPs are expected to be applied in the food and cosmetic industries as an effective emulsifier. However, studies on the effect of SNPs on skin cells and tissues have not been reported. Therefore, in the present study, we investigated the safety of SNPs in human keratinocytes (HaCaT) and fibroblasts (HDF), reconstructed 3D skin, micropig skin and human skin.

## 2. Results

### 2.1. Cytotoxicity of SNPs in HaCaT Cells and HDF Cells

**Figure 1:**
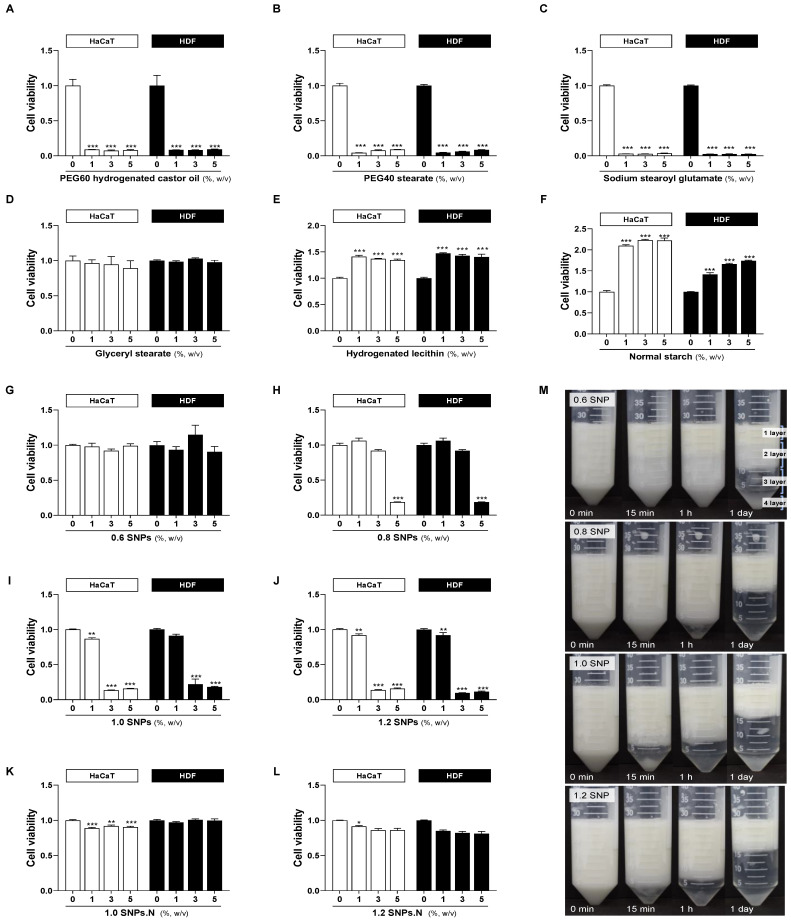
Cytotoxicity and emulsification of SNPs. Cytotoxicity of (**A**) PEG60 hydrogenated castor oil, (**B**) PEG40 stearate, (**C**) sodium stearoyl glutamate, (**D**) glyceryl stearate, (**E**) hydrogenated lecithin, (**F**) normal starch, (**G**) 0.6 SNPs, (**H**) 0.8 SNPs, (**I**) 1.0 SNPs, (**J**) 1.2 SNPs, (**K**) 1.0 SNPs.N and (**L**) 1.2 SNPs.N in both HaCaT cells and HDF cells. * *p* < 0.05, ** *p* < 0.01, *** *p* < 0.001 versus the 0% SNPs group. The experiment was repeated at least five times, and similar results were shown as mean ± S.E.M. (**M**) Image of Pickering emulsions stabilized by SNPs. The experiment was repeated at least three times, and similar results were shown as mean ± S.E.M.

To evaluate whether SNPs affect the viability of human skin cells, SNPs were dissolved in DMEM at 1–5% (*w*/*v*) and used to treat HaCaT cells and HDF cells. The commercially available emulsifiers PEG60, PEG40, SSG, GS, and HL were used as controls. The synthetic emulsifiers PEG60, PEG40, and SSG showed high cytotoxicity at all concentrations (Figure 1A–C). On the other hand, the naturally derived emulsifiers GS, HL, and normal starch had no toxicity affecting the viability of HaCaT cells and HDF cells at any tested concentration (Figure 1D–F). The 0.6 SNPs showed no cytotoxicity, but 0.8 SNPs significantly reduced the cell viability at the high concentration of 5% (Figure 1G,H). Notably, we identified cytotoxicity leading to decreased cell viability according to the amount of added acid. In particular, 1.0 SNPs and 1.2 SNPs showed strong cytotoxicity at more than 3% (Figure 1I,J). However, neutralized 1.0 SNPs.N and 1.2 SNPs.N did not show cytotoxicity (Figure 1K,L). Therefore, SNPs are considered to exhibit cytotoxicity due to the added acid, which can be overcome by a neutralization.

### 2.2. Emulsification of SNPs

A Pickering emulsion is a thermodynamically unstable system that, depending on the conservation period, causes phenomena such as flocculation, coalescence, creaming and sedimentation [10,14,15]. The separation of the emulsion due to density differences causes the cream layer to float. For 0.6 SNPs, oiling off was observed after 15 min of storage, and then the emulsion was clearly separated into an oil, emulsion, serum, and SNP sedimentation layers (from the first layer to the fourth layer) after storage for one day. However, stable emulsification was observed for SNPs supplemented with 0.8 mL or more of acid, and there was no significant difference related to acid amount (Figure 1M). Although creaming was observed after storage for one day, oiling-off was not observed for the emulsion stabilized by SNPs with 0.8 mL or more of acid. These data suggest that the addition of acid can increase the emulsion stability of SNPs.

### 2.3. The Effects of 1.0 SNPs and 1.0 SNPs.N in a 3D Cultured Skin Model

**Figure 2:**
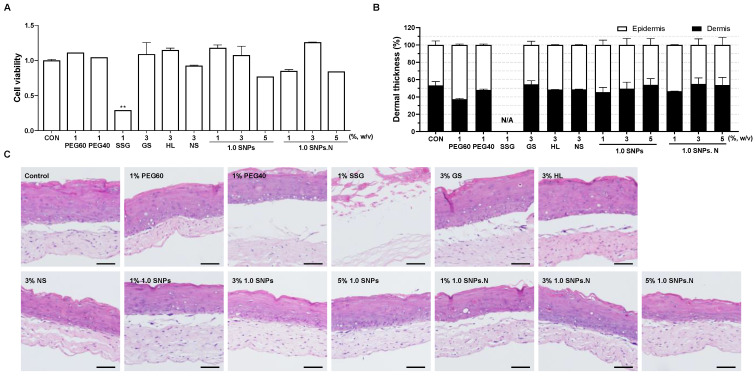
Cytotoxicity and histological analysis of SNPs and synthetic emulsifiers in 3D cultured human skin. (**A**) Cytotoxicity of 1.0 SNPs, 1.0 SNPs.N, and five commercial emulsifiers compared to normal cultured skin. The data are shown as the mean ± S.E.M. ** *p* < 0.01 versus the control group (CON). (**B**) Ratio graph of the epidermis and dermis visualized by hematoxylin and eosin (H & E) staining. (**C**) Images of H & E staining of the 3D cultured skin were acquired at 200× magnification, and representative images are shown; scale bar: 100 μm. The experiment was repeated at least three times and similar results were shown as mean ± S.E.M.

Based on the above results, we further investigated the safety of 1.0 SNPs and 1.0 SNPs.N in a 3D cultured skin model that replicates human skin. Cytotoxicity and histological analyses were performed to evaluate skin irritation with 1.0 SNPs and 1.0 SNPs.N. Commercially available emulsifiers, such as PEG60, PEG40, SSG, GS, and HL, were used as controls. The cytotoxicity of 1.0 SNPs and 1.0 SNPs.N in 3D cultured skin was investigated at 1–5%, and synthetic emulsifiers including PEG60 and naturally derived emulsifiers such as GS were used at 1% and 3%, respectively. Except for SSG, none of the emulsifiers, including SNPs, were toxic to the 3D cultured skin (Figure 2A). In addition, there was no significant change in the structure of the 3D cultured skin. Similar to the cytotoxicity results, none of the emulsifiers except SSG significantly changed the structure of the epidermis and the dermis (Figure 2B,C). Notably, SSG decomposed both the epidermis and the dermis, resulting in high cytotoxicity (Figure 2C). SSG is considered nonirritating and is frequently used in cosmetics and cleansers due to its softening and emulsifying properties. However, excessive use of emulsifiers can irritate the skin, and in fact, cases of allergic contact dermatitis caused by SSG have been reported [16]. Thus, we observed that SNPs were safer than synthetic emulsifiers, especially SSG, in 3D cultured human skin models.

### 2.4. The Permeation Effects of 1.0 SNPs and 1.0 SNPs.N in a Diffusion System

**Figure 3:**
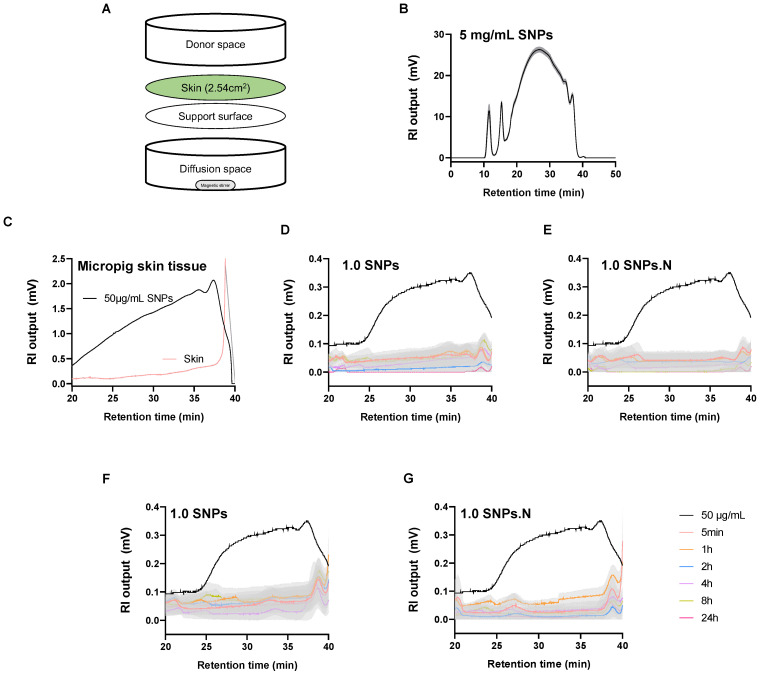
The permeation effect of 1.0 SNPs and 1.0 SNPs.N in a diffusion system. (**A**) Composition of the diffusion system and (**B**) HPSEC chromatogram of 5 mg/mL SNPs. The chromatogram of (**C**) micropig skin tissue, (**D**) diffused 1.0 SNPs and (**E**) diffused 1.0 SNPs.N with 50 μg/mL SNPs on micropig skin tissue. The chromatogram of (**F**) 1.0 SNPs and (**G**) 1.0 SNPs.N with 50 μg/mL SNPs on skin membranes. The experiment was repeated at least three times, and similar results were shown as mean ± S.E.M.

Next, we examined the skin permeability of SNPs. Some emulsifiers destroy the skin barrier and trigger allergies due to the permeation of antigens into the skin [17,18]. Recently, the increase in the number of patients with emulsifier contact dermatitis has become a problem [19,20]. Therefore, micropig skin tissues and start-M™ skin membranes were used to assess the permeability of SNPs in a diffusion system. Micropig tissues and start-M™ skin membranes were attached to the device, and diffusion samples were collected after exposure to SNPs for 5 min and 1, 2, 4, 8, and 24 h (Figure 3A). Saccharide derived from 5 mg/mL SNPs was detected within 20–40 min and analyzed under the same conditions (Figure 3B). No saccharide was detected in liquid nitrogen frozen micropig skin tissue (Figure 3C) or in time-dependent diffusion space samples (Figure 3D,E). Similar effects were observed in the start-M™ skin membrane (Figure 3F,G). These data indicate that the 1.0 SNPs and 1.0 SNPs.N do not permeate the skin or remain in the skin tissue.

### 2.5. The Irritation Effects of 1.0 SNPs and 1.0 SNPs.N on Human Skin

Based on the above results, primary irritation in human skin was evaluated. Patches loaded with 20 μL each of 1% 1.0 SNPs and 1% 1.0 SNPs.N were attached to human dorsal skin for 24 h. Stimulation levels were graded by expert visual assessment 30 min and 24 h after patch removal. As expected, none of the 33 subjects experienced any irritation, including erythema or itching. Because the visual evaluation grade was 0, the stimulation index was also calculated as 0.00 (Table 1). The 1.0 SNPs and 1.0 SNPs.N were found to belong in the “No irritation” category. Therefore, SNPs do not cause any real irritation to human skin.

## 3. Discussion

Due to their remarkable emulsification, dispersal and cleaning effects, synthetic emulsifiers are used in a variety of industries, including the cosmetics and food industries [21]. Synthetic emulsifiers have the advantage of being inexpensive and mass-produced. However, synthetic emulsifiers can irritate the skin, destroy its protective function and carry harmful substances into the skin [22]. In particular, synthetic emulsifiers present in almost all cosmetics, including soaps and creams, can cause contact dermatitis with erythema and itching [23,24,25]. Therefore, in this study, we investigated the safety of maize starch nanoparticles (SNPs) manufactured via acidic dry heat as effective emulsifiers. Notably, high emulsion stability was observed, with no significant differences except for the 0.6 SNPs. This suggests that SNPs prepared via modified acid hydrolysis are effective emulsifiers.

Human keratinocytes (HaCaT), which account for 80% of epidermal cells, form skin keratin and play an external barrier role [26]. Human fibroblasts (HDF) are known to form connective tissue in the skin, produce an extracellular matrix and play an important role in the wound healing process [27]. Therefore, it is possible to predict the effect of SNPs exposure on human skin in HaCaT cells and HDF cells. Many studies have reported the cytotoxicity of various emulsifiers, including PEG 7 glyceryl cocoate, and coemulsifiers, such as nanostructured lipid carriers, in HaCaT cells and HDF cells [28,29,30,31,32]. PEG 7 glyceryl cocoate, often used in cosmetics, including hair shampoo and rinse, was reported to cause mild irritation [28]. Sodium lauryl sulfate, a typical commercial emulsifier, also demonstrates cytotoxicity [29]. In addition, solid lipid nanoparticle-based stearic acid was reported to be cytotoxic [30]. In our study, SNPs manufactured with 0.8–1.2 mL of acid were cytotoxic to HaCaT cells and HDF cells. However, the neutralized 1.0 SNPs (1.0 SNPs.N) and 1.2 SNPs (1.2 SNPs.N) were confirmed to show reduced cytotoxicity. Thus, we found that maize starch converted to a nanosize by acidic dry heat followed by neutralization exhibits reduced cytotoxicity.

Moreover, we investigated the safety of 1.0 SNPs and 1.0 SNPs.N in 3D cultured skin because they formed a stable Pickering emulsion and were more economical than 1.2 SNPs. A 3D skin model similar to the human skin structure was used to assess structural safety and cytotoxicity [33]. As expected, treatment with 1–5% 1.0 SNPs.N left the skin structure unchanged, similar to normal 3D skin. Surprisingly, even 1.0 SNPs induced no significant changes. The Environmental Working Group (EWG), a nonprofit environmental citizenship organization in the United States, has established a safety standard for synthetic emulsifiers, with scores ranging from 0 (low risk) to 10 (high risk). EWG reported that synthetic emulsifiers, such as PEG60 hydrogenated castor oil (PEG60), PEG40 stearate (PEG40), sodium stearoyl glutamate (SSG), glyceryl stearate (GS), and hydrogenated lecithin (HL), are low-hazard (score under 3) ingredients. However, SSG completely decomposed the 3D cultured skin. SSG, classified as an anionic emulsifier, is often used in cosmetics, especially face cleansers [34]. Although research on the sensitivity of SSG is unclear, anionic emulsifiers are considered the harshest with regard to skin health compared to other emulsifiers [35,36]. The mechanisms of anionic emulsifiers in stratum corneum hydration are reported to be related to the irritation properties of anionic emulsifiers [37]. Considering these points, 1.0 SNPs and 1.0 SNPs.N are suggested to be safe, without any effect on the skin. However, PEG60 and PEG40 showed noncytotoxicity in 3D cultured skin, unlike in HaCaT cells and HDF cells. Indeed, 3D cultured skin is more likely to mimic the physiological response of skin than 2D cell cultures due to the interaction of the epidermis and dermis [38]. Nevertheless, 3D cultured skin tissue has a particular advantage in wound healing and absorption tests over cytotoxicity assays due to improved cell resistance to toxic agents [33,39]. Therefore, conflicting findings of cytotoxicity to 2D cells and 3D cultured skin have been reported, and these should be comprehensively considered [38,40].

The above results suggest that 1.0 SNPs and 1.0 SNPs.N are harmless to human skin ex vivo. Therefore, we performed a human skin irritation test. Dorsal skin exposed to the 1.0 SNPs and 1.0 SNPs.N for 24 h did not exhibit any irritation, including erythema and itching. One limitation of this study is that only 1.0 SNPs and 1.0 SNPs.N, not synthetic emulsifiers, were tested for skin irritation. Nevertheless, although various skin irritations caused by synthetic emulsifiers have been recently reported, both 1.0 SNPs and 1.0 SNPs.N were found to belong in the “No irritation” category. Based on this, we suggest that both 1.0 SNPs and 1.0 SNPs.N can be used as safe emulsifiers.

In addition, skin permeation and emulsifier residues can cause atopic dermatitis, allergies, mutations and chronic skin lesions [41,42]. Emulsifiers cause excessive solubilization of skin lipids, leading to itching, dryness, and inflammation via interaction with the stratum corneum [43]. In particular, synthetic emulsifiers cause skin cell apoptosis, cancer, and skin aging due to dysfunction of moisture retention and protein denaturation in the stratum corneum [44]. Therefore, emulsifiers induce emulsification of skin but should not permeate into the skin [45]. In vitro skin permeation tests can compare the absorption and permeability of a substance and can be a useful model for evaluating the permeation of human skin [46]. We found that 1.0 SNPs and 1.0 SNPs.N were not detected in the 24 h diffusion system of the micropig skin tissue and skin membrane, and no residue of the micropig skin tissue was detected. Therefore, lower skin permeabilities of 1.0 SNPs and 1.0 SNPs.N can be expected to result in less skin irritation and transdermal water loss (TEWL).

SNPs are manufactured with abundant and inexpensive natural starch, which is the most compelling reason for SNPs to be used as emulsifiers [8]. In previous studies, SNPs exhibited low particle aggregation due to their high absolute zeta potential in aqueous solutions [10]. In addition, SNPs can effectively encapsulate hydrophobic bioactive materials such as curcumin [46]. The properties of reported SNPs suggest that bioactive materials can be continuously delivered to drug targets. In this study, a modified acid hydrolysis method was used to increase production efficiency, and the 1.0 SNPs and 1.0 SNPs.N produced are effective in emulsification. Furthermore, without irritation that damages the skin, these nanoparticles can be used as safe natural emulsifiers in various fields, including cosmetics and topical pharmaceuticals. Thus, 1.0 SNPs and 1.0 SNPs.N can be effective and safe alternative emulsifiers for people with sensitive skin. Nevertheless, SNPs were less competitive in cost and emulsification than commercially available emulsifiers. Therefore, it is necessary to modify the manufacturing process of SNPs and evaluate their price competitiveness and excellent emulsification. Moreover, researching the skin protective or regenerative effects of SNPs could develop additional functions of SNPs and expand their application areas.

## 4. Materials and Methods

### 4.1. Preparation of Starch Nanoparticles (SNPs) and Emulsification Test

**Figure 4:**
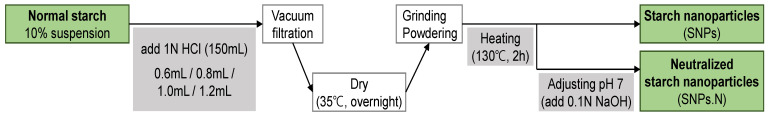
Diagram for preparing the SNPs.

SNPs were prepared via acidic dry heat treatment of normal maize starch as previously described by Choi et al. [13] and provided by the food processing laboratory at Kangwon National University (Prof. J.-Y. Kim) (Figure 4). The mean diameter of the studied SNPs was less than 30 nm, and the compositions are summarized in Table 2.

To conduct an emulsification test, each SNPs type (5%) was mixed with deionized distilled water (85% of the total volume) and homogenized at 20,000 rpm for 2 min at room temperature. Then, corn oil (10% of the total volume) was added, and the mixture was vortexed for 3 min. Afterward, stabilization of the 15 mL Pickering emulsions by homogenized SNP dispersions at 20,000 rpm for 6 min was observed.

### 4.2. Cell Culture and Cell Viability Analysis

Human epidermal keratinocytes (HaCaT) cells and human dermal fibroblasts (HDF) cells were provided by the Food Chemistry Laboratory at Kangwon National University (Prof. O.-H. Lee). Cells were cultured in Dulbecco’s modified Eagle’s medium (DMEM; Welgene, Gyeongsan, Korea) with 100 units/mL penicillin-streptomycin (P/S; Welgene, Gyeongsan, Korea) and 10% fetal bovine serum (FBS; Atlas Biologicals, Fort Collins, CO, USA) at 37 °C with 5% CO_2_, followed by subculture every three days [47,48]. Cell viability was measured to determine the cytotoxicity of SNPs by using MTT assays. HaCaT cells were treated with SNPs or commercially available emulsifiers for 24 h. The commercially available emulsifiers PEG60 hydrogenated castor oil (PEG60), PEG40 stearate (PEG40), sodium stearoyl glutamate (SSG), glyceryl stearate (GS), and hydrogenated lecithin (HL) were used as controls (Table 3). After incubation with 3-(4,5-dimethylthiazol-2-yl)-2,5-diphenyl tetrazolium bromide (MTT; Sigma Chemical Co., St. Louis, MO, USA) solution diluted 1:9 (5 mg/mL in PBS) at 37 °C for 4 h, purple formazan was formed in the cells. The solution in each well was completely removed, and then, the purple formazan crystals were dissolved in dimethyl sulfoxide (DMSO; Sigma Chemical Co., St. Louis, MO, USA) and isopropyl alcohol (Daejung, Seongnam, Korea) at 1:1 (100 μL/well). The optical density was measured at 540 nm by using a SpectraMax 190 microplate reader (Molecular Devices, San Jose, CA, USA).

### 4.3. Cell Viability Analysis in a Reconstituted 3D Human Skin Model

Reconstituted human skin (Neoderm-ED^TM^) was purchased from Tegoscience (Seoul, Korea). Neoderm-ED^TM^ is a commercially reconstituted human skin composed of keratinocytes and fibroblasts. The skin tissues were cultured in maintenance medium provided by the manufacturer and incubated at 37 °C with 5% CO_2_. The cytotoxicity of SNPs was measured by using MTT assays according to the manufacturer’s protocol [49]. Skin tissues were treated with SNPs or commercially available emulsifiers for 24 h. After incubation with MTT solution (25 mg/mL in PBS) diluted to 0.3 mg/mL in maintenance medium at 37 °C for 4 h, purple formazan was formed in the tissues. All tissues were collected and punched to a diameter of 5 mm, and then, the purple formazan crystals were dissolved in 1 mL of 0.04 N hydrochloric acid (HCl)-isopropyl alcohol for 4 h. The optical density was measured at 540 nm by using a SpectraMax 190 microplate reader (Molecular Devices, San Jose, CA, USA).

### 4.4. Histological Observation

After reconstituted 3D cultured skin was exposed to SNPs for 24 h, the sample was collected and fixed in 10% formalin solution at room temperature (20 ± 5 °C) and then embedded in paraffin [50]. Each section cut from the paraffin-embedded skin tissue was stained with hematoxylin and eosin (H & E). Images were obtained via light microscopy (Olympus, Tokyo, Japan), and histological analysis was conducted. The epidermal and dermal thicknesses were analyzed by observing the portion stained with H & E at 200× magnification.

### 4.5. Skin Permeation Test

The skin permeation test was based on the Ministry of Food and Drug Safety (MFDS) guideline for alternative animal test methods “in vitro skin absorption test” and the Organization for Economic Co-operation and Development (OECD) guideline for testing of chemicals, Section 4, TG 428 “Skin absorption: in vitro” [51]. The skin permeation test was performed by using a Franz diffusion cell system in micropig skin tissue (3 × 3 cm^2^, 300 μm, Apures, Pyeongtaek-si, Korea) and a start-M™ membrane (25 mm, Millipore, MA, USA). The tissues or the membranes were fixed on the support surface, and 12 mL of sterile distilled water in the diffusion space was stabilized at 32 ± 1 °C and 600 rpm. Then 1.0 SNPs and 1.0 SNPs.N were administered at 3% (*w*/*v* in distilled water) through the donor space, and 1.8 mL of each sample was collected from the diffusion space for analysis at 5 min and at 1, 2, 4, 8, and 24 h. The collected diffusion solution was subjected to high-pressure size exclusion chromatography (HPSEC) to determine the permeation of 1.0 SNPs and 1.0 SNPs.N.

### 4.6. High-Performance Size Exclusion Chromatography 

HPSEC is primarily employed for qualitative analysis of substances, and RI detectors can analyze and quantify components with limited or no UV absorption, such as alcohols, sugars, fatty acids, and macromolecules [52,53]. The HPSEC analysis was performed with a Water 1515 Isocratic System (Waters Corporation, MA, USA) and an RI detector (Waters Corporation, MA, USA). The samples were separated by using a Superdex™ 200 Increase 10/300 GL column (Cytiva, Marlborough, UK). The overall flow rate of the mobile phases was 0.5 mL/min, and the injection volume was 200 μL. The mobile phases used were water and 100 mM sodium chloride for diffusion space samples and micropig skin tissues, respectively. The collected diffusion space samples were diluted tenfold in distilled water, and the research micropig skin tissue was frozen in liquid nitrogen and then ground with a mortar.

### 4.7. Skin Irritation Assessment

Human irritation research was conducted in accordance with the integrated addendum to the ICH E6(R1) guideline for good clinical practice (E6(R2)) of the OATC Skin Clinical Trial Center, Inc. [54]. The skin irritation test was performed with the approval of the OATC institutional review board (OATC IRB) (2018071702-2108-HR-150-01). The degree of irritation caused by SNPs in the patch test was determined by expert visual evaluation. The study was explained to 33 Koreans aged 20–60 years with healthy skin who consented to participate. All skin irritations caused by 1% 1.0 SNPs and 1.0 SNPs.N were measured at baseline, after attachment of a patch for 24 h, and at 30 min and 24 h after removal of the patch. The irritation index was calculated by substituting the symptom-based score into the following formula (Table 4 and Table 5):

$$\mathrm{Skin}\;\mathrm{irritation}\;\mathrm{index}=\frac{\left[\left(\frac{\sum_{i=1}^{n}assessment\;grade}{n\;\left(number\;of\;subjects\right)}\right)30\;\min+\left(\frac{\sum_{i=1}^{n}assessment\;grade}{n\;}\right)24\;h\right]}{number\;of\;assessment}.$$

### 4.8. Statistical Analysis

All data were analyzed by using GraphPad Prism Version 8.0 (GraphPad, La Jolla, CA, USA). All measurements are expressed as the mean ± standard error of the mean (S.E.M.). All results were analyzed by using a Student–Newman–Keuls test for multiple comparisons after one-way analysis of variance (ANOVA) was performed. Significance was defined as *p* < 0.05.

## Figures and Tables

**Table 1:** Irritation evaluation of 1.0 SNPs and 1.0 SNPs.N on human skin. The test was repeated on 33 subjects with healthy skin, and similar results were shown.

Sample Name	Concentration	Visual Assessment Grade		Skin Irritation Index	Irritation Evaluation
		After 30 min	After 24 h		
1.0 SNPs	1% (*w*/*v*)	0	0	0.00	No irritation
1.0 SNPs.N		0	0	0.00	No irritation

**Table 2:** Compositions of the studied SNPs.

Sample Name	Vehicle	Acid Addition	Heating Time	Neutralization
Normal starch		0.0 mL	0 h	X
0.6 SNPs	Distilled water	0.6 mL	2 h	X
0.8 SNPs	Distilled water	0.8 mL	2 h	X
1.0 SNPs	Distilled water	1.0 mL	2 h	X
1.0 SNPs.N	Distilled water	1.0 mL	2 h	O
1.2 SNPs	Distilled water	1.2 mL	2 h	X
1.2 SNPs.N	30% EtOH	1.2 mL	3 h	O

**Table 3:** Table of used commercial emulsifiers as control.

Sample Name	Trade Name	Type
PEG60 hydrogenated castor oil	PEG60	Non-ionic synthetic emulsifier
PEG40 stearate	PEG40	Non-ionic synthetic emulsifier
Sodium stearoyl glutamate	SSG	Anionic synthetic emulsifier
Glyceryl stearate	GS	Naturally derived emulsifier
Hydrogenated lecithin	HL	Naturally derived emulsifier

**Table 4:** Visual assessment grade based on symptoms.

Symbol	Grade	Symptoms
	0	No reaction
+	1	Slight erythema, either spotty of diffuse
++	2	Moderate uniform erythema
+++	3	Intense erythema with edema
++++	4	Intense erythema with edema and vesicles

**Table 5:** Irritation evaluation range by skin irritation index.

Skin Irritation Index	Irritation Evaluation
0.00~0.25	No irritation
0.26~1.00	Mild irritation
1.01~2.50	Medium irritation
2.51~4.00	Strong irritation

## Sample Availability

Samples of the SNPs are available from the authors.

## Abbreviations

SNPs: starch nanoparticles; PEG60, polyoxyethylene 60 hydrogenated castor oil; PEG40, polyoxyethylene 40 stearate; SSG, sodium stearoyl glutamate; GS, glyceryl stearate; HL, hydrogenated lecithin.

## References

[1] Abismal B., Canselier J., Wilhelm A., Delmas H., Gourdon C. (1999). Emulsification by ultrasound: Drop size distribution and stability. Ultrason. Sonochem..

[2] Kale S.N., Deore S.L. (2017). Emulsion Micro Emulsion and Nano Emulsion: A Review. Syst. Rev. Pharm..

[3] Vieira M.V., Pastrana L.M., Fucinos P. (2020). Microalgae Encapsulation Systems for Food, Pharmaceutical and Cosmetics Applica-tions. Mar. Drugs.

[4] Tamang N., Shrestha P., Khadka B., Mondal M.H., Saha B., Bhattarai A. (2021). A Review of Biopolymers’ Utility as Emulsion Stabilizers. Polymers.

[5] Corazza M., Virgili A., Ricci M., Bianchi A., Borghi A. (2016). Contact Sensitization to Emulsifying Agents: An Underrated Issue?. Dermatitis.

[6] Moore D.J., Caso S., Vincent C., Ananthapadmanabhan K. (2004). pH induced alterations in stratum corneum properties. J. Am. Acad. Dermatol..

[7] Bornkessel A., Flach M., Elsner P., Fluhr J.W., Arens-Corell M. (2005). Functional assessment of a washing emulsion for sensitive skin: Mild impairment of stratum corneum hydration, pH, barrier function, lipid content, integrity and cohesion in a controlled washing test. Ski. Res. Technol..

[8] Zhu F. (2019). Starch based Pickering emulsions: Fabrication, properties, and applications. Trends Food Sci. Technol..

[9] Li C., Li Y., Sun P., Yang C. (2014). Starch nanocrystals as particle stabilisers of oil-in-water emulsions. J. Sci. Food Agric..

[10] Ko E.B., Kim J.-Y. (2021). Application of starch nanoparticles as a stabilizer for Pickering emulsions: Effect of environmental factors and approach for enhancing its storage stability. Food Hydrocoll..

[11] Fresco-Cala B., Cárdenas S. (2022). Advanced polymeric solids containing nano- and micro-particles prepared via emulsion-based polymerization approaches. A review. Anal. Chim. Acta.

[12] Angellier H., Choisnard L., Molina-Boisseau S., Ozil P., Dufresne A. (2004). Optimization of the preparation of aqueous suspensions of waxy maize starch nanocrystals using a response sur-face methodology. Biomacromolecules.

[13] Choi H.-D., Hong J.S., Pyo S.M., Ko E., Shin H.-Y., Kim J.-Y. (2020). Starch nanoparticles produced via acidic dry heat treatment as a stabilizer for a Pickering emulsion: Influence of the physical properties of particles. Carbohydr. Polym..

[14] Pirozzi A., Capuano R., Avolio R., Gentile G., Ferrari G., Donsì F. (2021). O/W Pickering Emulsions Stabilized with Cellulose Nanofibrils Produced through Different Mechanical Treatments. Foods.

[15] Chen L., Ao F., Ge X., Shen W. (2020). Food-Grade Pickering Emulsions: Preparation, Stabilization and Applications. Molecules.

[16] Pralong P., Dendooven E., Aerts O. (2022). Sodium stearoyl glutamate: Another amino acid alkyl amide sensitizer in cosmetics. Contact Dermat..

[17] Rahma A., Lane M.E. (2022). Skin Barrier Function in Infants: Update and Outlook. Pharmaceutics.

[18] Bárány E., Lindberg M., Lodén M. (2000). Unexpected skin barrier influence from nonionic emulsifiers. Int. J. Pharm..

[19] Navarro-Triviño F.J., Ruiz-Villaverde R. (2022). Allergic contact dermatitis in a psoriasis patient caused by cetylstearyl alcohol. Contact. Dermat..

[20] Nishioka K., Koizumi A., Takita Y. (2022). Seven cases of contact dermatitis due to stearyl alcohol contained in topical medications. J. Dermatol..

[21] McClements D.J., Gumus C.E. (2016). Natural emulsifiers—Biosurfactants, phospholipids, biopolymers, and colloidal particles: Molecu-lar and physicochemical basis of functional performance. Adv. Colloid Interf. Sci..

[22] Williams R.M. (2006). Makeup goes organic. Townsend Lett. Exam. Altern. Med..

[23] Uter W., Werfel T., White I.R., Johansen J.D. (2018). Contact Allergy: A Review of Current Problems from a Clinical Perspective. Int. J. Environ. Res. Public Health.

[24] Hannuksela M., Kousa M., Pirilä V. (1976). Contact sensitivity to emulsifiers. Contact. Dermat..

[25] Hannuksela M. (1988). Skin contact allergy to emulsifiers. Int. J. Cosmet. Sci..

[26] He B., Wang Y., Peng D., Wang L., Dong Z. (2015). Screening of HaCaT Clones for CCL20 Gene Knockout and Preliminary Exploration of Gene-Targeting Vector Transfection Approaches in this Cell Line. Med. Sci. Monit. Basic Res..

[27] Bautista-Hernández L.A., Gómez-Olivares J.L., Buentello-Volante B., Bautista-De Lucio V.M. (2017). Fibroblasts: The unknown sentinels eliciting immune responses against microorganisms. Eur. J. Microbiol. Immunol..

[28] Fiume M.M., Bergfeld W.F., Belsito D.V., Hill R.A., Klaassen C.D., Liebler D.C., Marks J.J.G., Shank R.C., Slaga T.J., Snyder P.W. (2020). Safety Assessment of PEGylated Alkyl Glycerides as Used in Cosmetics. Int. J. Toxicol..

[29] Benavides T., Mitjans M., Martínez V., Clapés P., Infante M.R., Clothier R.H., Vinardell M.P. (2004). Assessment of primary eye and skin irritants by in vitro cytotoxicity and phototoxicity models: An in vitro ap-proach of new arginine-based surfactant-induced irritation. Toxicology.

[30] Weyenberg W., Filev P., Van den Plas D., Vandervoort J., De Smet K., Sollie P., Ludwig A. (2007). Cytotoxicity of submicron emulsions and solid lipid nanoparticles for dermal application. Int. J. Pharm..

[31] Wilhelm K.P., Böttjer B., Siegers C.P. (2001). Quantitative assessment of primary skin irritants in vitro in a cytotoxicity model: Comparison with in vivo human irritation tests. Br. J. Dermatol..

[32] Korting H., Herzinger T., Hartinger A., Kerscher M., Angerpointner T., Maibach H. (1994). Discrimination of the irritancy potential of surfactants in vitro by two cytotoxicity assays using normal human keratinocytes, HaCaT cells and 3T3 mouse fibroblasts: Correlation with in vivo data from a soap chamber assay. J. Dermatol. Sci..

[33] Teimouri A., Yeung P., Agu R. (2018). 2D vs. 3D Cell Culture Models for In Vitro Topical (Dermatological) Medication Testing. Cell Culture.

[34] Effendy I., Maibach H.I. (1995). Surfactants and experimental irritant contact dermatitis. Contact Dermat..

[35] Morris S.A., Ananthapadmanabhan K.P., Kasting G.B. (2019). Anionic Surfactant–Induced Changes in Skin Permeability. J. Pharm. Sci..

[36] Wilhelm K.P., Cua A.B., Wolff H.H., Maibach H.I. (1993). Surfactant-induced stratum corneum hydration in vivo: Prediction of the irritation potential of anionic surfac-tants. J. Investig. Derm..

[37] Barua N., Huang L., Li C., Yang Y., Luo M., Wei W.I., Wong K.T., Lo N.W.S., Kwok K.O., Ip M. (2022). Comparative Study of Two-Dimensional (2D) vs. Three-Dimensional (3D) Organotypic Kertatinocyte-Fibroblast Skin Models for Staphylococcus aureus (MRSA) Infection. Int. J. Mol. Sci..

[38] Sun T., Jackson S., Haycock J., MacNeil S. (2006). Culture of skin cells in 3D rather than 2D improves their ability to survive exposure to cytotoxic agents. J. Biotechnol..

[39] Breslin S., O’Driscoll L. (2016). The relevance of using 3D cell cultures, in addition to 2D monolayer cultures, when evaluating breast cancer drug sensitivity and resistance. Oncotarget.

[40] Gloor M. (2004). How do dermatological vehicles influence the horny layer?. Ski. Pharm. Physiol.

[41] Zesch A. (1988). Adverse reactions of externally applied drugs and inert substances. Dermatosen Beruf und Umwelt. Occup. Environ..

[42] Chen Y., Qiao F., Fan Y., Han Y., Wang Y. (2017). Interactions of Cationic/Anionic Mixed Surfactant Aggregates with Phospholipid Vesicles and Their Skin Penetration Ability. Langmuir.

[43] Salvioni L., Morelli L., Ochoa E., Labra M., Fiandra L., Palugan L., Prosperi D., Colombo M. (2021). The emerging role of nanotechnology in skincare. Adv. Colloid Interf. Sci..

[44] James-Smith M.A., Hellner B., Annunziato N., Mitragotri S. (2010). Effect of Surfactant Mixtures on Skin Structure and Barrier Properties. Ann. Biomed. Eng..

[45] Abd E., Yousuf S.A., Pastore M.N., Telaprolu K., Mohammed Y., Namjoshi S., Grice J.E., Roberts M.S. (2016). Skin models for the testing of transdermal drugs. Clin. Pharmacol. Adv. Appl..

[46] Miskeen S., An Y.S., Kim J.-Y. (2021). Application of starch nanoparticles as host materials for encapsulation of curcumin: Effect of citric acid modification. Int. J. Biol. Macromol..

[47] Yang Y.-I., Woo J.-H., Seo Y.-J., Lee K.-T., Lim Y., Choi J.-H. (2016). Protective Effect of Brown Alga Phlorotannins against Hyper-inflammatory Responses in Lipopolysaccharide-Induced Sepsis Models. J. Agric. Food Chem..

[48] Huh M.-I., Kim M.-S., Kim H.-K., Lim J.O. (2014). Effect of conditioned media collected from human amniotic fluid-derived stem cells (hAFSCs) on skin regeneration and photo-aging. Tissue Eng. Regen. Med..

[49] Kang Y.-M., Seo M.-G., Lee K.-Y., An H.-J. (2020). Potential of Extracts of Yin-Tonic Herbal Medicine in Skin Cell and Human Skin Equivalent. Evid. Based Complement. Altern. Med..

[50] Kim K., Kim J., Kim H., Sung G. (2021). Effect of α-Lipoic Acid on the Development of Human Skin Equivalents Using a Pumpless Skin-on-a-Chip Model. Int. J. Mol. Sci..

[51] Guth K., Schäfer-Korting M., Fabian E., Landsiedel R., van Ravenzwaay B. (2015). Suitability of skin integrity tests for dermal absorption studies in vitro. Toxicol. Vitr..

[52] Yao Y., Guiltinan M.J., Thompson D.B. (2005). High-performance size-exclusion chromatography (HPSEC) and fluorophore-assisted carbohydrate electrophoresis (FACE) to describe the chain-length distribution of debranched starch. Carbohydr. Res..

[53] Wade J.H., Bailey R.C. (2014). Refractive index-based detection of gradient elution liquid chromatography using chip-integrated mi-croring resonator arrays. Anal. Chem..

[54] Yoo M.A., Kim S.H., Han H.S., Byun J.W., Park K.H. (2022). The effects of wearing a face mask and of subsequent moisturizer use on the characteristics of sensitive skin. Ski. Res. Technol..

